# Masking Ability of Various Metal Complexing Ligands at 1.0 mM Concentrations on the Potentiometric Determination of Fluoride in Aqueous Samples

**DOI:** 10.1155/2020/6683309

**Published:** 2020-12-18

**Authors:** Sakuni M. De Silva, Samitha Deraniyagala, Janitha K. Walpita, Indira Jayaweera, Saranga Diyabalanage, Asitha T. Cooray

**Affiliations:** ^1^Instrument Centre, Office of the Dean, Faculty of Applied Sciences, University of Sri Jayewardenepura, Gangodawila, Nugegoda, Sri Lanka; ^2^Department of Chemistry, Faculty of Applied Sciences, University of Sri Jayewardenepura, Gangodawila, Nugegoda, Sri Lanka; ^3^Ecosphere Resilience Research Center, Faculty of Applied Sciences, University of Sri Jayewardenepura, Gangodawila, Nugegoda, Sri Lanka

## Abstract

Fluoride is a common anion present in natural waters. Among many analytical methods used for the quantification of fluoride in natural waters, potentiometric analysis is one of the most widely used methods because of minimum interferences from other ions commonly present in natural waters. The potentiometric analysis requires the use of ionic strength adjusting buffer abbreviated as TISAB to obtain accurate and reproducible data. In most of the reported literature, higher concentrations of strong metal chelating ligands are used as masking agents generally in the concentration range of 1.0 to 0.01 M. In the present study, effectiveness of the masking agents, phosphate, citrate, CDTA ((1,2-cyclohexylenedinitrilo)tetraacetic acid), EDTA (ethylenediaminetetraacetic acid) HE-EDTA ((hydroxyethyl)ethylenediaminetriacetic acid)), triethanolamine, and tartaric acid at 1.0 mM in TISAB solutions was investigated. The experimental data were compared with a commercially available WTW 140100 TISAB solution as the reference buffer. According to the experimental data, the reference buffer always produced the highest fluoride concentrations and the measured fluoride concentrations were in the range of 0.611 to 1.956 mg/L. Out of all the masking agents investigated, only CDTA performed marginally well and approximately a quarter of the samples produced statistically comparable data to the reference buffer. All the other masking agents produced significantly low concentrations compared to the reference buffer. The most probable reasons for the underestimation of fluoride concentrations could be shorter decomplexing time and lower masking agent concentrations.

## 1. Introduction

Fluoride is a ubiquitous anion present in natural waters. It has been long recognized for its beneficial impacts on oral health [1]. The optimal and beneficial concentration of fluoride for human health generally falls within a narrow concentration range of 0.5 to 1.5 mg/L [1]. According to international drinking water standards, fluoride concentrations below 0.5 mg/L results in dental caries while fluoride content above 1.5 mg/L causes dental and skeletal fluorosis [2]. However, the maximum recommended fluoride concentration in drinking water varies significantly among various countries [1]. Presence of excess fluoride in surface and groundwater has been a crucial health problem in South Asian countries including Sri Lanka [3, 4], India [5–7], Pakistan [8], Indonesia [9], and African countries including Ethiopia [10], Sudan [11], Tanzania [12], Kenya [13], Uganda [14], Nigeria [15], and Ghana [16]. Consumption of water contaminated with excessive amounts of fluoride for a prolonged period of time increases the risk of fluorosis, a health condition which is easily recognized by dental mottling and skeletal manifestations [17]. In addition, fluoride has been suggested as a risk factor for the endemic chronic kidney disease of unknown etiology in Sri Lanka where the prevalence rate varies between 8 and 16% in the dry climate zone [18–20]. The majority of Sri Lankans living in dry zone are suffering from endemic fluorosis as a result of consuming fluoride rich groundwater from dug and deep wells [2, 3, 21].

One of the most effective strategies to mitigate the adverse health effects of fluoride is to identify the fluoride hotspots and make available necessary water purification techniques to affected communities. Therefore, accurate quantification of fluoride in drinking water sources is essential [6, 21, 22]. In addition, fluoride also has been quantified in other materials including vinegar [23], powdered milk [24], glass [25], phosphate rocks [26], and tooth paste [27–30]. Quantification of fluoride is also essential to evaluate the efficiency of naval fluoride removal materials developed recently such as trimetallic composites [31, 32]. There are several analytical methods available to quantify fluoride in a wide variety of sample matrixes. Some of the most commonly used methods include colorimetry [3, 21, 33, 34], chromatography [35], and potentiometry [16, 20, 24, 25, 31, 32, 36, 37]. Among these methods, potentiometry that uses a fluoride ion selective electrode (F-ISE) is a popular choice mainly due to field portability for *in-situ* analysis, ease of operation, higher selectivity, broad linear range, and minimum inferences from common anions and cations in the sample matrix [38, 39]. One of the main requirements of the potentiometric method is the availability of a suitable total ionic strength adjusting buffer (abbreviated as TISAB) to obtain accurate results [38]. Unlike the other methods used for fluoride quantification, potentiometric methods measure the activity of fluoride ions, not its concentration [38, 39]. In order to keep the fluoride ion activity coefficient constant independent of the ionic strength of samples, all the samples and calibration standards are buffered to a high ionic strength using a NaCl solution. This solution also contains an acetic acid-acetate pH buffer to maintain the pH between pH 5.0 and 5.5 that minimizes interferences from OH^−^ ions and also prevents the formation of HF. In addition, most of the TISAB solutions also contain strong metal chelating ligands or masking agents that could break strong metal-fluoride complexes mostly with polyvalent cations like Al^3+^ and Fe^3+^ that enable the quantification of total fluoride instead of free fluoride concentration [39, 40].

In general, commercially available TISAB solutions formulated as TISAB I, II, III, and IV [41] are generally added to the sample in 1 : 1 ratio (10 mL: 10 mL). The average cost for 500 mL of commercially available TISABs is priced between USD 150 and 200 in 2020. Due to the higher cost of commercially available TISAB solutions, there is a tendency to use lab prepared TISAB solutions in fluoride analysis [29, 42]. It has been shown that the masking agents used in the TISAB solution could significantly affect the accuracy of the method [25, 26, 43, 44]. Unfortunately, in some of the published literature, the authors have neglected to disclose the actual composition of TISAB if they were prepared in the lab or at least to provide brand name if a commercially available one was used in analysis [16, 20, 24, 45, 46]. In most cases, TISAB composition optimization for fluoride analysis has been carried out using standard fluoride solutions spiking with possible interfering ions such as Al^3+^, Fe^3+^, Mg^2+^, and Ca^2+^. In addition, the concentration of masking agents is usually in the range of 1.0 to 0.01 M [40, 43]. In the reported literature, individual masking agents [26, 39, 40, 42, 44] or a combination of masking agents [23, 25, 40] have been used in TISAB solutions.

The main objective of this research is to assess the effect of the masking agents at relatively low concentration of 1.0 mM in TISAB solutions on the potentiometric determination of fluoride in aqueous samples. In this study, a commercially available WTW branded TISAB solution (Model 140100) was used as the reference TISAB solution. The fluoride concentrations of sixty (60) natural water samples were determined using the reference and eight (08) lab prepared TISAB solutions containing different types of masking agents. Finally, the fluoride concentrations were statistically analyzed to evaluate the effectiveness of the individual masking agents used in lab prepared TISAB solutions.

## 2. Materials and Methods

### 2.1. Materials and Reagents

All chemicals were of analytical reagent grade and were used without any further purification. Double distilled water was used throughout the study for solution preparation. Standard fluoride solutions were prepared using NaF (s). A commercially available WTW branded TISAB (Model 140100) was used as the reference solution. Lab prepared TISAB solutions contain 1.0 M NaCl, 0.10 M acetic acid/sodium acetate pH buffer solution adjusted to pH 5.5 and a 1.0 mM masking agent. The masking agents used in this study were sodium phosphate, sodium citrate, CDTA (trans-1, 2-cyclohexanediamine-n, n, n′, n′-tetraacetic acid monohydrate), EDTA disodium salt (disodium ethylenediaminetetraacetate dihydrate), HE-EDTA (hydroxyethyl)ethylenediaminetriacetic acid), triethanolamine (2, 2′, 2″-nitrilotriethanol), and tartaric acid (2, 3-dihydroxysuccinic acid). One buffer solution was prepared without any metal chelating ligand and one buffer containing only NaCl.

### 2.2. Instrumentations

The fluoride concentration of the water samples was measured using a WTW model F106667 fluoride selective electrode coupled to a WTW 340i multi-ion meter. Concentration of the metal ions was determined using an Agilent 4210 MP-AES instrument and the conductivity of the samples was measured using a EUTECH CyberScan Con 11 portable conductivity meter.

### 2.3. Determination of Fluoride Ion Content

Sixty (60) water samples were collected from different areas of Sri Lanka into precleaned polypropylene bottles. Samples were stored at 4°C and transported back to lab and analyzed for fluoride within 72 h.

The WTW 340i multi-ion meter was calibrated using fluoride standard solutions of 0.1, 1.0, and 5.0 mg/L concentrations and the TISAB of interest. The fluoride content in samples was recorded according to the guidelines provided by the instrument manufacturer. In brief, an aliquot of 10.0 mL of the sample or the calibration standard was mixed with 10.0 mL of the selected TISAB solution in a 50 mL HDPE beaker. The mixture was placed on a magnetic stirrer with the probe and a PTFE coated magnet bar. The content was gently stirred, and the fluoride concentration was recorded or the instrument was calibrated once a stable reading was established. All the analyses were duplicated.

### 2.4. Determination of Metal Ion Concentrations

Out of sixty (60) water samples, twenty (20) samples were selected for metal analysis and the concentrations of Ca, Mg, Al, and Fe were determined. The selected samples were first acidified to pH < 2 using high purity HNO_3_ and then filtered using 0.22 *μ*m Nylon syringe filters. The analysis was carried out using MP-AES (Agilent 4210 MP-AES) instrument. Standard calibration curves were prepared for Ca, Mg, and Fe (from 500 mg/L multielement mixture) at 0.50, 0.75, 1.00, 1.50, and 2.00 ppm and for Al (from 1000 mg/L standard) at 0.25, 0.50, 0.75, 1.00, and 1.50 mg/L. The analytical wavelengths used for the analysis for the metals Al, Fe, Mg, and Ca were 396.152 nm, 358.119 nm, 280.27 nm, and 616.217 nm, respectively.

### 2.5. Statistical Analysis

The data for fluoride analysis were expressed as the average value measured through F-ISE. Statistical analysis was conducted using MINITAB 17.

## 3. Results and Discussion

### 3.1. Fluoride Concentration in the Samples

The concentrations of masking agents in the TISAB solutions sometimes vary from study to study. Often, 0.03% w/v sodium citrate has been used as the masking agent in TISAB solutions [26, 27, 29, 39, 40, 44]; however, much higher concentrations, has also been used [23, 40, 43]. The most commonly used CDTA concentration is 0.4% w/v [40, 42]. In this study, the concentration of all the masking agents is 1.0 mM.

The fluoride concentration data of the samples measured with the TISAB solution containing only NaCl was not in good quality mainly because it took a long time to establish a stable reading. As a result, it was difficult to calibrate the instrument using standard fluoride solutions and TISAB containing only NaCl. In addition, relative standard deviation (RSD) of the duplicate measurements was very high (up to 25 % in some cases) compared to less than 5 % RSD with most of other TISAB buffer solutions. Therefore, the fluoride data gathered from TISAB containing only NaCl was not included and was not statistically analyzed. The fluoride concentrations of 60 water samples were tabulated in the Table 1. All the raw fluoride concentration data is available as supplementary material.

**Figure 1:**
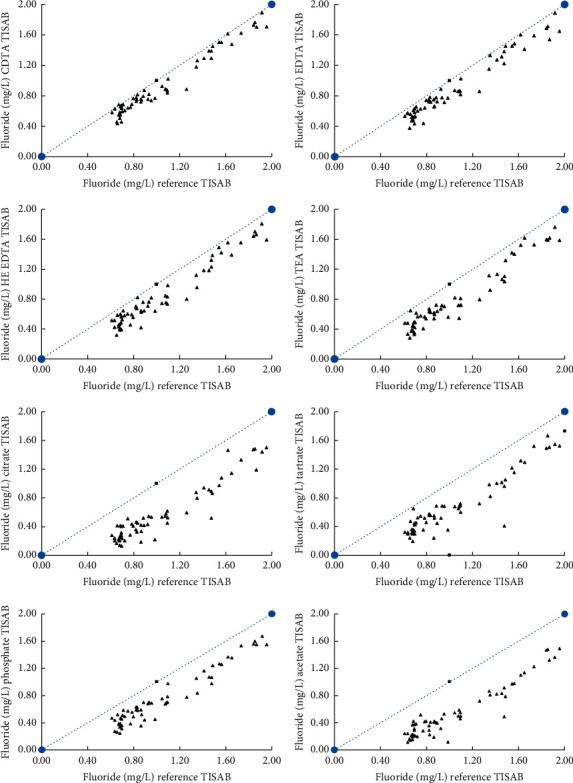
Graphical representation of the fluoride concentrations determined by the lab prepared TISAB solutions with respect to reference buffer solution.

The fluoride concentrations were in the range of 0.611 to 1.956 mg/L when the samples were analyzed using the reference TISAB solution and electrical conductivity of the samples was in the range of 53.5 to 481 *μ*S/cm. According to experimental data, the highest fluoride concentration in samples was always measured using the reference TISAB buffer, while the lowest fluoride concentrations were measured with TISAB buffers containing either citrate or acetate as the masking agent. The fluoride concentrations determined by the lab prepared TISAB solutions with respect to reference buffer solution are graphically presented in Figure 1. According to the data presented in Table 1 and Figure 1, it appears that CDTA, EDTA, HE-EDTA, and TEA are much better masking agents at 1.0 mM concentrations than citrate, tartrate, phosphate, and acetate. All the masking agents used in this study form stable complexes with metal ions including the ones that were analyzed in this study. It should be noted that the formation constants of metal-ligand complexes are strongly dependent on ionic strength (*I*) of the solution. For an example, formation constants (log *K*) of Ca-EDTA (CaY^2−^) and Mg-EDTA (MgY^2−^) complexes are 12.4 and 10.6 at *I* = 0 and 10.7 and 8.69 at *I* = 0.1, respectively [47, 48]. Therefore, it is expected that formation constants to be smaller at higher ionic strengths used in TISAB solutions. As a result, it is expected to have a low tendency to form metal-ligand complexes at higher ionic strengths. This could be the reason for using much higher masking agents up to 1.0 M in reported literature [43].

For about a quarter of samples, reference buffer and CDTA-TISAB produced statistically comparable fluoride concentrations when the mean fluoride concentrations were evaluated with the Student's *t*-test. The percentage difference of fluoride concentrations between the reference and lab prepared TISAB solutions was calculated and it is tabulated in Table 2.

The data presented in Table 2 was totally unexpected due to the fact that TISAB solutions with 1.0 mM citrate (∼0.03% w/v) has been previously used in literature [26, 27, 29, 39, 40, 44] and it is one of the commonly used TISAB solutions for fluoride analysis. On the other hand, CDTA concentration used in many TISAB solutions is 0.01 M (∼0.4% w/v) [40, 42] which is ten times more concentrated than the CDTA concentration used in this study. The main reason to use 1.0 mM masking agents in TISAB solutions was to evaluate their effectiveness at relatively low concentrations as a mean of reducing the cost of sample analysis and reducing the environmental pollution as per Green Chemistry principles. To our knowledge, this is the only recent study that has used real natural water samples and relatively low masking agent concentrations to study the effectiveness of low concentration masking agents in TISAB solutions.

### 3.2. Effect of Metal Ions on the Analysis of Fluoride

Polyvalent metal ions such as Fe^3+^, Al^3+^, Mg^2+^, and Ca^2+^ play an important role in the potentiometric analysis of fluoride because these metal ions make stable complexes with fluoride and it is necessary to break them using a suitable masking agent before the analysis. In general, CDTA has been used as a masking agent for Al^3+^ and Fe^3+^, while citrate has been used as a masking agent for Ca^2+^ and Mg^2+^. At the pH of TISAB of 5.50, it is expected that phosphate, citrate, CDTA, EDTA, and tartaric acid to be present as H_2_PO_4_^−^, HCit^2−^, H_2_CDTA^2−^, H_2_Y^2−^(H_2_EDTA^2−^), and tartrate, respectively. The formation constants of these species with the metal ions Mg^2+^, Ca^2+^, Fe^3+^, and Al^3+^ are available in literature [48, 49]. Most of the available formation constants have been determined at low ionic strengths in the range of 0 to 0.1 mol/L and, therefore, not directly applicable to predict chemical reactions that take place in higher ionic strength solutions such as in TISAB solutions with an ionic strength -approximately 1 mol/L. Nevertheless, the formation constants are helpful to predict whether a particular masking agent can dissociate metal-fluoro complexes. Some of the formation constants available in literature are presented in Table 3 according to [48, 49]. According to the formation constants given in Table 3, it is clear that CDTA and EDTA have the ability to break down metal-fluoride complexes and release free fluoride ions to the solution. It should be noted that ionic strength,pH of the medium, temperature, presence of other chelating ligands, such as natural organic matter, and the presence of polyvalent metal ions also affect the breakdown of metal-fluoro complexes by the making agents.

It has been reported that metal ions Ca^2+^, Mg^2+^, Fe^3+^, and Al^3+^ could interfere with the fluoride analysis at relatively higher concentrations [40]. Therefore, total metal concentrations of Ca, Mg, Fe, and Al measured in the selected twenty samples are presented in Table 4.

The concentration of metals was in the range of 0.05 to 1.09 mg/L for Al, 0.03 to 7.48 mg/L for Fe, 0.03 to 9.96 mg/L for Mg, and 1.93 to 19.06 mg/L for Ca. Though the formation constants of metal-ligand complexes are used to explain the masking action of ligands, the effectiveness of masking agents also depends on kinetics of the reaction. According to Nicolson and Duff [40], preferred maximum and minimum decomplexing times are 24 h and 20 min, respectively. In this study, samples mixed with TISAB buffers were left to stand for about 5 mins before the measurements were taken once a stable reading was established, usually in less than 3 mins.

It appears that the shorter decomplexing time and relatively low concentrations of masking agents at 1.0 mM might have contributed to the underestimation of fluoride in the tested samples with compared to reference buffer solution. A brand-new F-ISE was used in the study and the instrument calibration with individual TISAB solutions was always successful except for TISAB containing only NaCl. Therefore, electrode condition and the instrument calibration could not have contributed to the underestimation of fluoride concentration by lab-made TISAB solution. The fluoride concentrations in the samples were also analyzed by a YSI 9500 Photometer and Palintest (YPM179) reagents and the results are presented in Table 1. Palintest method is based on zirconyl chloride and eriochrome cyanine *R* colorimetric method. A careful look at the experimental data reveals that the photometric data is much more statistically comparable with citrate-TISAB data than reference TISAB data. Photometric data was always lower than the reference TISAB data; however, 25% of citrate-TISAB data was within ± 5.0 % of photometric data.

## 4. Conclusions

In this study, the effectiveness of the masking agents, phosphate, citrate, CDTA, EDTA, HE-EDTA, triethanolamine, and tartaric acid at 1.0 mM in TISAB solutions for the use of potentiometric determination of fluoride in natural water samples were investigated. The experimental data were compared with a commercially available WTW 140100 TISAB solution which was used as the reference buffer. According to the experimental data, reference buffer always produced the highest fluoride concentrations and the measured fluoride concentrations were in the range of 0.611 to 1.956 mg/L. CDTA-TISAB performed marginally well and only about quarter of the samples produced statistically comparable data to the reference buffer. All the other masking agents produced significantly low concentrations compared to reference buffer. The most probable reasons for the underestimation of fluoride concentrations could be shorter decomplexing time and lower masking agent concentrations. However, lab-prepared TISAB solutions such as CDTA-TISAB can be identified as a probable cost-effective alternative to the commercially available TISAB solutions in order to obtain accurate results for fluoride ion quantifying procedures.

## Figures and Tables

**Table 1:** Fluoride content of 60 water samples measured by F-ISE with different masking agents.

Sample ID	Mean fluoride concentration (mg L^−1^)										EC (*μ*S/cm)
	Ref.	CDTA	EDTA	HE-EDTA	TEA	Citrate	Tartrate	Phosphate	Acetate	Photometer	
1	1.842	1.730	1.689	1.645	1.595	1.475	1.493	1.556	1.469	N/A	126.4
2	1.619	1.614	1.603	1.558	1.524	1.467	1.317	1.364	1.098	1.30	177.8
3	1.868	1.703	1.542	1.669	1.620	1.193	1.506	1.548	1.321	N/A	149.1
4	1.543	1.503	1.456	1.492	1.422	0.975	1.218	1.266	0.969	1.03	165.1
5	1.734	1.625	1.592	1.556	1.526	1.332	1.518	1.530	1.221	N/A	127.5
6	1.563	1.503	1.486	1.426	1.403	1.083	1.153	1.259	0.974	1.15	288.0
7	1.486	1.450	1.455	1.388	1.317	0.863	1.052	1.240	0.784	0.91	181.2
8	1.854	1.763	1.715	1.703	1.593	1.485	1.662	1.598	1.475	N/A	221.0
9	1.916	1.892	1.889	1.809	1.762	1.440	1.545	1.672	1.360	N/A	156.3
10	1.476	1.390	1.383	1.323	1.104	0.522	0.962	1.067	0.486	0.96	231.0
11	0.864	0.758	0.753	0.645	0.542	0.188	0.238	0.352	0.186	0.24	117.9
12	0.796	0.764	0.743	0.559	0.544	0.408	0.456	0.486	0.419	0.40	78.9
13	0.686	0.659	0.611	0.598	0.487	0.415	0.648	0.467	0.420	0.42	53.3
14	0.693	0.637	0.522	0.486	0.496	0.403	0.445	0.458	0.374	0.42	92.6
15	1.352	1.260	1.334	0.959	0.921	0.800	0.817	0.837	0.808	0.82	79.9
16	0.749	0.641	0.627	0.605	0.577	0.512	0.545	0.571	0.513	0.53	97.0
17	0.695	0.457	0.437	0.392	0.355	0.319	0.338	0.359	0.309	0.34	115.2
18	1.261	0.885	0.859	0.801	0.797	0.593	0.717	0.775	0.720	0.73	134.6
19	1.344	1.179	1.154	1.121	1.114	0.876	0.983	1.055	0.862	0.94	194.5
20	1.652	1.478	1.413	1.391	1.618	1.147	1.294	1.354	1.137	1.29	191.2
21	0.722	0.607	0.604	0.582	0.566	0.206	0.521	0.494	0.196	0.49	282.0
22	0.679	0.504	0.524	0.469	0.441	0.142	0.194	0.244	0.149	0.16	339.0
23	1.476	1.291	1.225	1.230	1.039	0.889	0.409	0.977	0.912	0.91	128.6
24	1.455	1.390	1.314	1.187	1.065	0.916	1.018	1.069	0.831	1.00	261.0
25	0.636	0.628	0.569	0.424	0.333	0.206	0.318	0.269	0.111	0.26	188.5
26	0.696	0.642	0.633	0.576	0.420	0.132	0.297	0.307	0.209	0.29	342.0
27	1.098	1.019	1.029	0.986	0.720	0.528	0.601	0.976	0.495	0.52	296.0
28	0.986	0.767	0.773	0.640	0.561	0.220	0.352	0.448	0.115	0.30	154.7
29	0.954	0.739	0.716	0.820	0.702	0.533	0.680	0.686	0.529	0.58	79.0
30	1.956	1.706	1.651	1.596	1.587	1.503	1.521	1.548	1.489	N/A	196.6
31	0.611	0.578	0.535	0.519	0.482	0.282	0.320	0.467	0.239	0.39	382
32	0.636	0.632	0.576	0.518	0.482	0.244	0.299	0.437	0.237	0.25	87.8
33	0.654	0.457	0.375	0.319	0.282	0.169	0.241	0.260	0.161	0.13	65.1
34	0.924	0.756	0.893	0.712	0.672	0.431	0.518	0.678	0.315	0.44	179.4
35	0.701	0.592	0.533	0.527	0.493	0.237	0.425	0.511	0.210	0.39	83.1
36	0.694	0.675	0.583	0.565	0.329	0.280	0.422	0.453	0.289	0.33	88.8
37	0.671	0.686	0.475	0.437	0.396	0.221	0.311	0.376	0.208	0.24	97.2
38	0.682	0.552	0.483	0.387	0.447	0.264	0.358	0.387	0.225	0.30	116.0
39	0.679	0.542	0.475	0.401	0.376	0.253	0.318	0.370	0.369	0.30	111.1
40	0.659	0.436	0.562	0.588	0.648	0.415	0.472	0.516	0.142	0.44	55.6
41	0.671	0.550	0.521	0.456	0.377	0.224	0.360	0.389	0.219	0.23	276.0
42	0.678	0.583	0.521	0.433	0.378	0.204	0.298	0.336	0.192	0.27	70.3
43	1.046	0.924	0.873	0.745	0.820	0.535	0.679	0.753	0.547	0.59	72.0
44	1.076	0.889	0.859	0.848	0.720	0.547	0.652	0.677	0.498	0.61	73.6
45	0.709	0.683	0.657	0.646	0.618	0.410	0.453	0.583	0.383	0.43	62.4
46	0.884	0.793	0.778	0.760	0.723	0.521	0.685	0.697	0.400	0.67	401.0
47	0.784	0.687	0.642	0.596	0.566	0.439	0.518	0.555	0.410	0.49	353.0
48	1.083	0.880	0.863	0.757	0.548	0.583	0.679	0.694	0.586	0.67	84.9
49	0.771	0.650	0.441	0.456	0.408	0.284	0.355	0.385	0.281	0.28	132.4
50	0.821	0.777	0.753	0.706	0.672	0.357	0.561	0.587	0.288	0.54	318.0
51	0.864	0.719	0.656	0.420	0.620	0.424	0.547	0.589	0.409	0.52	481.0
52	0.933	0.820	0.779	0.765	0.718	0.544	0.686	0.699	0.457	0.62	382.0
53	0.822	0.727	0.722	0.698	0.633	0.464	0.577	0.629	0.357	0.57	252.0
54	1.095	0.866	0.821	0.739	0.721	0.453	0.690	0.701	0.457	0.61	231.0
55	1.412	1.294	1.276	1.187	1.133	0.944	1.003	1.160	0.819	0.95	149.3
56	0.835	0.817	0.718	0.826	0.626	0.453	0.556	0.578	0.408	0.54	153.2
57	1.094	0.836	0.865	0.833	0.812	0.617	0.718	0.783	0.549	0.68	251.0
58	0.828	0.752	0.781	0.679	0.634	0.326	0.552	0.627	0.223	0.34	263.0
59	0.892	0.870	0.726	0.648	0.638	0.421	0.446	0.439	0.395	0.38	116.0
60	0.868	0.745	0.666	0.626	0.596	0.337	0.518	0.518	0.422	0.40	57.6

N/A = not analyzed; Ref. = WTW TISAB solution; TEA = triethanolamine; EC = electrical conductivity.

**Table 2:** The percentage difference of fluoride concentrations between the reference and selected TISAB solutions.

TISAB combination	The percentage difference			
	<5%	5.01–10%	10.01–15%	>15%
	Percentage of samples come under each % difference			
Ref: CDTA	26.7	20.0	21.7	31.7
Ref.: EDTA	8.3	25.0	16.7	50.0
Ref.: HE-EDTA	5.0	11.7	8.3	75.0
Ref.: TEA	3.3	5.0	11.7	80.0
Ref.: citrate	0.0	0.0	1.17	98.0
Ref.: tartrate	0.0	1.7	3.3	95.0
Ref.: phosphate	0.0	0.0	6.7	93.3
Ref.: acetate	0.0	0.0	0.0	100

**Table 3:** Stability constants log (*K*) values of masking agents for different ligands adapted from [48, 49].

Ligand	Stability constants log(*K*) values of masking agents at *t* = 25°C and *I* = 0 mol/L			
	Ca^2+^	Mg^2+^	Al^3+^	Fe^3+^
F^−^	10.4	8.2	16.7	13.7
	CaF_2_	MgF_2_	AlF_3_	FeF_3_

OH^−^	5.19	11.16	27.0	42.7
	Ca(OH)_2_	Mg(OH)_2_	Al(OH)_3_	Fe(OH)_3_

CDTA	15.0	12.8	22.1	32.6
	(CaCDTA^2−^)	(MgCDTA^2−^)	(AlCDTA^−^)	(FeCDTA^−^)

EDTA (Y^4−^)	12.4	10.6	18.9	27.7
	(CaY^2−^)	(MgY^2−^)	(AlY^−^)	(FeY^−^)

H_2_PO_4_^−^	21.0	20.0	N/A	23.9

Hydrogencitrate	2.81	2.42	N/A	13.5

Acetate	1.2	1.3	2.4	4.0
	[CaCH_3_COO]^+^	[MgCH_3_COO]^+^	[AlCH_3_COO]^2+^	[FeCH_3_COO]^2+^

Tartrate	2.98	2.35	N/A	N/A

N/A: not available.

**Table 4:** The total metal concentration of Al, Fe, Mg, and Ca in selected water samples.

Sample ID	Al content (mg/L)	Fe content (mg/L)	Mg content (mg/L)	Ca content (mg/L)
1	0.97	4.14	1.08	10.17
3	0.96	6.30	1.38	8.69
4	1.09	7.48	1.50	5.92
5	0.78	7.40	1.75	6.04
6	0.82	7.18	9.96	19.06
7	0.67	5.40	2.07	11.34
9	0.05	0.82	0.20	3.54
11	0.30	2.31	1.54	17.03
13	0.27	2.39	1.85	7.45
14	0.18	1.92	0.39	3.78
15	0.44	2.52	0.18	3.65
16	0.38	0.66	0.33	3.27
20	0.05	0.38	0.14	2.98
21	0.24	0.56	0.86	6.82
23	0.10	0.45	0.29	3.23
24	0.08	0.41	0.37	6.60
26	0.11	0.30	0.37	5.66
30	0.30	0.12	0.13	6.53
41	0.25	0.03	0.03	5.07
43	0.27	0.51	0.10	1.93

## Data Availability

All the raw data are included within the manuscript. Additional information can be obtained through the corresponding author.

## Supplementary Materials

Supplementary Materials The fluoride concentration of the water samples was measured using a WTW model F106667 fluoride selective electrode coupled to a WTW 340i multi-ion meter. Lab prepared TISAB solutions contain 1.0 M NaCl, 0.10 M acetic acid/sodium acetate pH buffer solution adjusted to pH 5.5, and a 1.0 mM masking agent. The masking agents used in this study were sodium phosphate, sodium citrate, CDTA (trans-1, 2-cyclohexanediamine- n, n, n′, n′-tetraacetic acid monohydrate), EDTA disodium salt (disodium ethylenediaminetetraacetate dihydrate), HE-EDTA (hydroxyethyl)ethylenediaminetriacetic acid), triethanolamine (2, 2′, 2″-nitrilotriethanol), and tartaric acid (2, 3-dihydroxysuccinic acid).
