# Supplementary material for: Masking Ability of Various Metal Complexing Ligands at 1.0 mM Concentrations on the Potentiometric Determination of Fluoride in Aqueous Samples
Source: J Anal Methods Chem. 2020 Dec 18;2020:6683309. doi: 10.1155/2020/6683309 (PMC7762639; doi:10.1155/2020/6683309)
Supplement: Supplementary Materials — The fluoride concentration of the water samples was measured using a WTW model F106667 fluoride selective electrode coupled to a WTW 340i multi-ion meter. Lab prepared TISAB solutions contain 1.0 M NaCl, 0.10 M acetic acid/sodium acetate pH buffer solution adjusted to pH 5.5, and a 1.0 mM masking agent. The masking agents used in this study were sodium phosphate, sodium citrate, CDTA (trans-1, 2-cyclohexanediamine- n, n, n′, n′-tetraacetic acid monohydrate), EDTA disodium salt (disodium ethylenediaminetetraacetate dihydrate), HE-EDTA (hydroxyethyl)ethylenediaminetriacetic acid), triethanolamine (2, 2′, 2″-nitrilotriethanol), and tartaric acid (2, 3-dihydroxysuccinic acid). [file 6683309.f1.pdf]

The fluoride concentration of the water samples was measured using a WTW model F106667 fluoride selective electrode coupled to a WTW 340i multi-ion meter. Lab prepared TISAB solutions contain 1.0 M NaCl, 0.10 M acetic acid / sodium acetate pH buffer solution adjusted to pH 5.5 and a 1.0 mM masking agent. The masking agents used in this study were sodium phosphate, sodium citrate, CDTA (trans-1,2-cyclohexanediamine- n,n,n',n'-tetraacetic acid monohydrate), EDTA disodium salt (disodium ethylenediaminetetraacetatedihydrate), HE-EDTA (Hydroxyethyl)ethylenediaminetriacetic acid), triethanol amine (2,2',2''-Nitrilotriethanol), and tartaric acid (2,3-dihydroxysuccinic acid)

	Reference			CDTA			EDTA			HE EDTA			Tri-Ethanol ammine			Citrate			Tartrate			Phosphate buffer			Acetate buffer			
Sample	Trial 1	Trial 2	Average	Trial 1	Trial 2	Average	Trial 1	Trial 2	Average	Trial 1	Trial 2	Averag	Trial 1	Trial 2	Averag	Trial 1	Trial 2	Average	Trial 1	Trial 2	Averag	Trial 1	Trial 2	Average	Trial 1	Trial 2	Average	Photometer
1	1.852	1.832	1.842	1.714	1.746	1.730	1.679	1.698	1.689	1.640	1.649	1.645	1.594	1.595	1.595	1.488	1.462	1.475	1.509	1.476	1.493	1.556	1.555	1.556	1.475	1.462	1.469	N/A
2	1.623	1.615	1.619	1.618	1.610	1.614	1.591	1.615	1.603	1.574	1.542	1.558	1.519	1.529	1.524	1.482	1.451	1.467	1.322	1.312	1.317	1.372	1.356	1.364	1.112	1.084	1.098	1.300
3	1.855	1.881	1.868	1.722	1.684	1.703	1.536	1.548	1.542	1.671	1.667	1.669	1.603	1.637	1.620	1.202	1.184	1.193	1.511	1.501	1.506	1.554	1.542	1.548	1.329	1.312	1.321	N/A
4	1.526	1.560	1.543	1.516	1.490	1.503	1.468	1.444	1.456	1.502	1.481	1.492	1.433	1.410	1.422	0.982	0.968	0.975	1.210	1.225	1.218	1.271	1.261	1.266	0.977	0.961	0.969	1.030
5	1.756	1.712	1.734	1.641	1.609	1.625	1.591	1.592	1.592	1.545	1.567	1.556	1.534	1.518	1.526	1.342	1.321	1.332	1.519	1.516	1.518	1.515	1.545	1.530	1.226	1.216	1.221	N/A
6	1.554	1.572	1.563	1.494	1.512	1.503	1.478	1.494	1.486	1.431	1.421	1.426	1.415	1.391	1.403	1.090	1.076	1.083	1.154	1.151	1.153	1.260	1.257	1.259	0.980	0.968	0.974	1.150
7	1.488	1.484	1.486	1.462	1.438	1.450	1.469	1.441	1.455	1.378	1.398	1.388	1.311	1.323	1.317	0.870	0.856	0.863	1.061	1.043	1.052	1.247	1.232	1.240	0.790	0.778	0.784	0.910
8	1.842	1.866	1.854	1.755	1.771	1.763	1.719	1.711	1.715	1.709	1.697	1.703	1.598	1.588	1.593	1.498	1.472	1.485	1.677	1.647	1.662	1.602	1.594	1.598	1.481	1.468	1.475	N/A
9	1.921	1.911	1.916	1.902	1.882	1.892	1.886	1.892	1.889	1.818	1.800	1.809	1.775	1.749	1.762	1.455	1.425	1.440	1.553	1.537	1.545	1.679	1.664	1.672	1.366	1.354	1.360	N/A
10	1.451	1.501	1.476	1.397	1.383	1.390	1.375	1.390	1.383	1.335	1.311	1.323	1.095	1.113	1.104	0.531	0.513	0.522	0.964	0.959	0.962	1.073	1.061	1.067	0.499	0.473	0.486	0.960
11	0.871	0.856	0.864	0.762	0.754	0.758	0.763	0.742	0.753	0.656	0.634	0.645	0.566	0.518	0.542	0.193	0.182	0.188	0.246	0.229	0.238	0.362	0.342	0.352	0.190	0.181	0.186	0.240
12	0.810	0.782	0.796	0.772	0.756	0.764	0.726	0.760	0.743	0.569	0.549	0.559	0.557	0.531	0.544	0.414	0.401	0.408	0.461	0.451	0.456	0.499	0.472	0.486	0.425	0.413	0.419	0.400
13	0.693	0.679	0.686	0.662	0.656	0.659	0.618	0.604	0.611	0.605	0.591	0.598	0.507	0.466	0.487	0.416	0.413	0.415	0.654	0.642	0.648	0.472	0.461	0.467	0.413	0.426	0.420	0.420
14	0.691	0.695	0.693	0.647	0.626	0.637	0.518	0.526	0.522	0.501	0.471	0.486	0.499	0.493	0.496	0.412	0.394	0.403	0.453	0.437	0.445	0.466	0.449	0.458	0.380	0.367	0.374	0.420
15	1.366	1.338	1.352	1.264	1.256	1.260	1.346	1.322	1.334	0.967	0.951	0.959	0.931	0.911	0.921	0.813	0.787	0.800	0.822	0.812	0.817	0.849	0.824	0.837	0.812	0.804	0.808	0.820
16	0.738	0.760	0.749	0.653	0.629	0.641	0.634	0.620	0.627	0.596	0.614	0.605	0.598	0.555	0.577	0.523	0.501	0.512	0.553	0.537	0.545	0.579	0.563	0.571	0.510	0.516	0.513	0.530
17	0.698	0.691	0.695	0.461	0.452	0.457	0.428	0.446	0.437	0.397	0.386	0.392	0.366	0.343	0.355	0.324	0.313	0.319	0.346	0.329	0.338	0.348	0.370	0.359	0.312	0.306	0.309	0.340
18	1.249	1.272	1.261	0.892	0.878	0.885	0.866	0.852	0.859	0.810	0.792	0.801	0.802	0.791	0.797	0.601	0.584	0.593	0.721	0.713	0.717	0.788	0.761	0.775	0.726	0.713	0.720	0.730
19	1.338	1.350	1.344	1.182	1.176	1.179	1.169	1.138	1.154	1.129	1.112	1.121	1.120	1.108	1.114	0.891	0.861	0.876	0.990	0.976	0.983	1.066	1.043	1.055	0.866	0.858	0.862	0.940
20	1.648	1.656	1.652	1.469	1.486	1.478	1.423	1.403	1.413	1.399	1.383	1.391	1.624	1.611	1.618	1.152	1.141	1.147	1.302	1.285	1.294	1.366	1.342	1.354	1.143	1.130	1.137	1.290
21	0.722	0.721	0.722	0.612	0.601	0.607	0.614	0.593	0.604	0.568	0.596	0.582	0.578	0.553	0.566	0.211	0.201	0.206	0.529	0.513	0.521	0.501	0.486	0.494	0.205	0.186	0.196	0.490
22	0.671	0.687	0.679	0.512	0.495	0.504	0.509	0.539	0.524	0.473	0.465	0.469	0.451	0.431	0.441	0.161	0.123	0.142	0.203	0.185	0.194	0.231	0.257	0.244	0.152	0.145	0.149	0.160
23	1.482	1.470	1.476	1.296	1.285	1.291	1.245	1.204	1.225	1.228	1.232	1.230	1.048	1.029	1.039	0.913	0.865	0.889	0.413	0.404	0.409	0.981	0.972	0.977	0.922	0.902	0.912	0.910
24	1.429	1.480	1.455	1.392	1.388	1.390	1.327	1.301	1.314	1.190	1.184	1.187	1.119	1.011	1.065	0.920	0.912	0.916	1.024	1.011	1.018	1.075	1.062	1.069	0.840	0.821	0.831	1.000
25	0.642	0.630	0.636	0.616	0.639	0.628	0.574	0.564	0.569	0.435	0.413	0.424	0.344	0.321	0.333	0.210	0.201	0.206	0.321	0.314	0.318	0.271	0.267	0.269	0.109	0.113	0.111	0.260
26	0.708	0.684	0.696	0.655	0.629	0.642	0.641	0.624	0.633	0.577	0.575	0.576	0.427	0.413	0.420	0.116	0.148	0.132	0.305	0.289	0.297	0.310	0.304	0.307	0.204	0.214	0.209	0.290
27	1.101	1.094	1.098	1.026	1.012	1.019	1.001	1.056	1.029	0.991	0.981	0.986	0.727	0.713	0.720	0.516	0.539	0.528	0.608	0.594	0.601	0.984	0.967	0.976	0.507	0.482	0.495	0.520
28	0.998	0.974	0.986	0.772	0.761	0.767	0.783	0.762	0.773	0.659	0.620	0.640	0.580	0.542	0.561	0.226	0.213	0.220	0.346	0.358	0.352	0.452	0.443	0.448	0.102	0.128	0.115	0.300
29	0.952	0.956	0.954	0.746	0.731	0.739	0.728	0.703	0.716	0.829	0.811	0.820	0.688	0.716	0.702	0.542	0.523	0.533	0.672	0.687	0.680	0.695	0.676	0.686	0.534	0.524	0.529	0.580
30	1.961	1.950	1.956	1.712	1.700	1.706	1.660	1.642	1.651	1.607	1.584	1.596	1.598	1.576	1.587	1.512	1.494	1.503	1.529	1.512	1.521	1.554	1.541	1.548	1.501	1.476	1.489	N/A
31	0.618	0.604	0.611	0.593	0.562	0.578	0.543	0.526	0.535	0.525	0.512	0.519	0.499	0.464	0.482	0.296	0.267	0.282	0.326	0.314	0.320	0.473	0.461	0.467	0.247	0.231	0.239	0.390
32	0.636	0.636	0.636	0.636	0.628	0.632	0.586	0.566	0.576	0.522	0.513	0.518	0.493	0.471	0.482	0.249	0.238	0.244	0.309	0.289	0.299	0.442	0.431	0.437	0.241	0.233	0.237	0.250
33	0.647	0.660	0.654	0.446	0.467	0.457	0.382	0.367	0.375	0.325	0.312	0.319	0.297	0.266	0.282	0.172	0.165	0.169	0.246	0.235	0.241	0.268	0.251	0.260	0.168	0.154	0.161	0.130
34	0.912	0.936	0.924	0.764	0.748	0.756	0.907	0.879	0.893	0.712	0.712	0.712	0.692	0.651	0.672	0.439	0.423	0.431	0.521	0.515	0.518	0.683	0.673	0.678	0.311	0.318	0.315	0.440
35	0.703	0.698	0.701	0.588	0.596	0.592	0.542	0.524	0.533	0.508	0.545	0.527	0.499	0.487	0.493	0.246	0.228	0.237	0.428	0.422	0.425	0.516	0.506	0.511	0.216	0.203	0.210	0.390
36	0.699	0.689	0.694	0.670	0.680	0.675	0.597	0.568	0.583	0.574	0.556	0.565	0.336	0.321	0.329	0.295	0.264	0.280	0.428	0.416	0.422	0.456	0.449	0.453	0.298	0.279	0.289	0.330
37	0.688	0.654	0.671	0.683	0.689	0.686	0.493	0.456	0.475	0.431	0.443	0.437	0.405	0.387	0.396	0.228	0.213	0.221	0.316	0.305	0.311	0.388	0.364	0.376	0.214	0.202	0.208	0.240
38	0.681	0.683	0.682	0.566	0.537	0.552	0.494	0.471	0.483	0.394	0.380	0.387	0.452	0.442	0.447	0.269	0.259	0.264	0.356	0.359	0.358	0.392	0.382	0.387	0.216	0.234	0.225	0.300
39	0.689	0.669	0.679	0.548	0.536	0.542	0.481	0.469	0.475	0.412	0.389	0.401	0.386	0.365	0.376	0.264	0.241	0.253	0.324	0.312	0.318	0.375	0.364	0.370	0.372	0.366	0.369	0.300
40	0.646	0.672	0.659	0.435	0.436	0.436	0.569	0.555	0.562	0.591	0.584	0.588	0.647	0.649	0.648	0.415	0.415	0.415	0.472	0.472	0.472	0.516	0.516	0.516	0.142	0.142	0.142	0.440
41	0.681	0.660	0.671	0.566	0.533	0.550	0.529	0.513	0.521	0.466	0.446	0.456	0.408	0.346	0.377	0.235	0.213	0.224	0.365	0.354	0.360	0.392	0.386	0.389	0.224	0.213	0.219	0.230
42	0.662	0.694	0.678	0.599	0.567	0.583	0.529	0.512	0.521	0.446	0.419	0.433	0.383	0.373	0.378	0.213	0.194	0.204	0.307	0.289	0.298	0.340	0.331	0.336	0.197	0.186	0.192	0.270

43	1.040	1.051	1.046	0.919	0.929	0.924	0.884	0.861	0.873	0.861	0.629	0.745	0.827	0.812	0.820	0.542	0.527	0.535	0.687	0.671	0.679	0.765	0.740	0.753	0.551	0.542	0.547	0.590
44	1.081	1.070	1.076	0.901	0.876	0.889	0.862	0.855	0.859	0.855	0.841	0.848	0.729	0.710	0.720	0.556	0.537	0.547	0.656	0.648	0.652	0.682	0.671	0.677	0.509	0.487	0.498	0.610
45	0.701	0.717	0.709	0.688	0.677	0.683	0.672	0.642	0.657	0.671	0.621	0.646	0.624	0.612	0.618	0.416	0.403	0.410	0.465	0.441	0.453	0.587	0.579	0.583	0.399	0.366	0.383	0.430
46	0.896	0.872	0.884	0.801	0.784	0.793	0.792	0.764	0.778	0.765	0.754	0.760	0.734	0.712	0.723	0.528	0.513	0.521	0.695	0.675	0.685	0.708	0.686	0.697	0.404	0.395	0.400	0.670
47	0.796	0.771	0.784	0.691	0.682	0.687	0.646	0.638	0.642	0.604	0.588	0.596	0.578	0.554	0.566	0.446	0.431	0.439	0.522	0.513	0.518	0.561	0.548	0.555	0.415	0.405	0.410	0.490
48	1.089	1.077	1.083	0.892	0.868	0.880	0.882	0.843	0.863	0.768	0.745	0.757	0.564	0.532	0.548	0.599	0.566	0.583	0.686	0.671	0.679	0.704	0.683	0.694	0.590	0.581	0.586	0.670
49	0.787	0.755	0.771	0.657	0.642	0.650	0.455	0.426	0.441	0.463	0.449	0.456	0.416	0.400	0.408	0.292	0.276	0.284	0.366	0.344	0.355	0.394	0.376	0.385	0.289	0.272	0.281	0.280
50	0.813	0.829	0.821	0.781	0.773	0.777	0.764	0.741	0.753	0.711	0.700	0.706	0.682	0.662	0.672	0.366	0.348	0.357	0.572	0.549	0.561	0.593	0.581	0.587	0.295	0.280	0.288	0.540
51	0.849	0.879	0.864	0.725	0.712	0.719	0.668	0.643	0.656	0.427	0.413	0.420	0.627	0.613	0.620	0.434	0.413	0.424	0.556	0.538	0.547	0.598	0.579	0.589	0.416	0.401	0.409	0.520
52	0.942	0.924	0.933	0.826	0.813	0.820	0.791	0.766	0.779	0.763	0.766	0.765	0.724	0.712	0.718	0.552	0.536	0.544	0.695	0.677	0.686	0.709	0.689	0.699	0.464	0.450	0.457	0.620
53	0.815	0.828	0.822	0.741	0.713	0.727	0.726	0.718	0.722	0.708	0.688	0.698	0.645	0.621	0.633	0.472	0.456	0.464	0.582	0.572	0.577	0.633	0.625	0.629	0.363	0.350	0.357	0.570
54	1.084	1.105	1.095	0.886	0.846	0.866	0.829	0.813	0.821	0.754	0.724	0.739	0.726	0.716	0.721	0.457	0.448	0.453	0.699	0.681	0.690	0.708	0.694	0.701	0.462	0.451	0.457	0.610
55	1.419	1.404	1.412	1.301	1.286	1.294	1.285	1.267	1.276	1.189	1.185	1.187	1.139	1.127	1.133	0.959	0.928	0.944	1.011	0.994	1.003	1.169	1.151	1.160	0.825	0.813	0.819	0.950
56	0.842	0.828	0.835	0.822	0.811	0.817	0.710	0.726	0.718	0.830	0.821	0.826	0.633	0.619	0.626	0.467	0.438	0.453	0.568	0.543	0.556	0.583	0.572	0.578	0.411	0.405	0.408	0.544
57	1.099	1.088	1.094	0.845	0.826	0.836	0.876	0.854	0.865	0.844	0.821	0.833	0.816	0.808	0.812	0.622	0.612	0.617	0.722	0.713	0.718	0.793	0.773	0.783	0.556	0.541	0.549	0.680
58	0.820	0.836	0.828	0.761	0.742	0.752	0.792	0.769	0.781	0.689	0.668	0.679	0.641	0.627	0.634	0.338	0.313	0.326	0.565	0.539	0.552	0.636	0.618	0.627	0.229	0.216	0.223	0.340
59	0.899	0.885	0.892	0.885	0.855	0.870	0.739	0.713	0.726	0.667	0.628	0.648	0.632	0.644	0.638	0.429	0.413	0.421	0.456	0.435	0.446	0.446	0.432	0.439	0.406	0.383	0.395	0.380
60	0.891	0.845	0.868	0.755	0.734	0.745	0.676	0.655	0.666	0.638	0.613	0.626	0.589	0.603	0.596	0.348	0.325	0.337	0.508	0.528	0.518	0.522	0.513	0.518	0.428	0.416	0.422	0.400
